# Evaluation of Different Outlier Detection Methods for GPS Networks

**DOI:** 10.3390/s8117344

**Published:** 2008-11-17

**Authors:** Ertan Gökalp, Oğuz Güngör, Yüksel Boz

**Affiliations:** 1 Department of Geodesy and Photogrammetry, Karadeniz Technical University, Trabzon, 61080 Turkey; E-Mails: gokalp@ktu.edu.tr; ogungor@ktu.edu.tr; 2 Department of Geodesy and Photogrammetry, Hacettepe University, Ankara, Turkey; E-Mail: boz.1@osu.edu

**Keywords:** Robust estimation, Fuzzy logic, GPS, Statistical test, Data Snooping, Membership value

## Abstract

GPS (Global Positioning System) devices can be used in many applications which require accurate point positioning in geosciences. Accuracy of GPS decreases due to outliers resulted from the errors inherent in GPS observations. Several approaches have been developed to detect outliers in geodetic observations. It is important to determine which method is most effective at distinguishing outliers from normal observations. This paper investigates the behavior of conventional statistical test methods (Data Snooping (DS), Tau and t tests), some robust methods (Andrews's M-Estimation, Huber's M-Estimation, Tukey's M-Estimation, Danish Method, Yang-I M-Estimation, Yang-II M-Estimation, and fuzzy logic method in detection of outliers for three GPS networks having different characteristics. Test results are evaluated and the performances of different methods are presented quantitatively.

## 1. Introduction

Geoscience applications such as determination of crustal movements, deformations and landslides require accurate point positioning. GPS can be used as a tool in these applications due to its accurate point positioning ability. The 3-D coordinates of the GPS satellites are known precisely with respect to an Earth fixed coordinate system. GPS receivers measure code and phase to every satellite. For accurate positioning, absolute positioning is not used in GPS. Instead, baselines connecting control points are determined. This is also called relative positioning. In relative positioning (at millimeter level), at least two GPS receivers are occupied at two control points (position of one control point is known) and the code and phase observations to at least four GPS satellites are measured simultaneously. These measurements are repeated for a certain period of time which leads to redundant observations. If coordinates of one of the control points are known, the coordinates of the second point are determined using the baseline components. For example, let *A* is a control point whose coordinates are known, and *B* is the point whose coordinates are to be determined. The baseline components of these two points are measured using GPS receivers and the *X*, *Y*, *Z* coordinates of point *B* are obtained as:

(1) $$\begin{array}{l} X_{B}=X_{A}+\Delta X_{A-B}\\ Y_{B}=Y_{A}+\Delta Y_{A-B}\\ Z_{B}=Z_{A}+\Delta Z_{A-B} \end{array}$$

**Figure 1.**
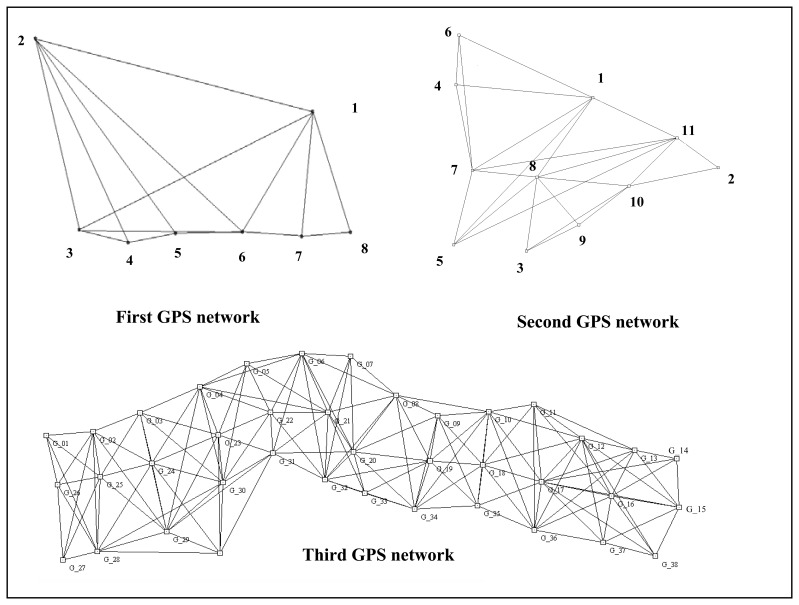
First, second and third GPS networks.

GPS networks are made up of baselines (see Figure 1), their baseline components *ΔX*, *ΔY*, and *ΔZ* are taken as observations, and estimated coordinates are obtained by Least Squares (LS) adjustment. However, some observations can be outliers and they may reduce the accuracy of the network; therefore, outliers should be detected and eliminated from the adjustment not to affect the rest of the observations.

In geodetic observations, errors can be in different magnitudes and have different characteristics depending on the surveyor, surveying equipment and environmental conditions. These errors yield differences in the magnitudes of the observations and are categorized as systematic, gross, and random errors. The effects of systematic and gross errors on the observations must be eliminated; however, it is not possible to remove random errors. Random errors are assumed to follow a normal distribution and are always present in the observations. Observations having random errors that deviate from normal distribution are called ‘outliers’.

The preliminary studies were implemented using two models. In the ‘mean-shift’ model, which is used by conventional methods, the outliers are detected step-by-step using statistical tests. In every step an observation is detected as an outlier and removed from the observation set. In the conventional methods of Data Snooping (DS), Tau, and t tests, there is a disadvantage since these methods remove outlying baselines which in turn deteriorate the shape of the network. A normal observation may be detected as an outlier or an outlying observation may be perceived as a normal observation because of the existing outliers in the observation set. Another model to detect outliers is ‘variance-inflation’ model which is used in robust estimations [1]. This model was developed to eliminate the effects of the outliers in the adjustment model. Any outlier is not removed from the network; however, the weights of the observations are changed after iterations. The weights of the outliers are reduced even to zero while the weights of the normal observations are kept unchanged during the iterations. The most important point in robust methods is defining the most acceptable critical value for weight functions. This critical value can be computed or selected as a constant value. In other works [2] this scheme of computing the robust estimator by iteratively reweighting has been replaced by the solution of a global optimization problem in the space of unknowns. The fuzzy logic approach uses both the fuzzy set and the statistical test theory to determine outliers. Contrary to the conventional methods, no observation is removed from the observation set. In addition, a more accurate decision is given for the observations close to the critical value.

In this paper, outliers in GPS networks are detected using different outlier detection and robust estimation methods, and the performances of these methods are investigated to evaluate their behaviors and similarities in detection of existing outliers.

## 2. Methods

### 2.1 Conventional Statistical Test Methods

These methods are based on the assumption that only one observation can be detected as an outlier in each iteration step of the adjustment. Outlying observation is identified using statistical test theory. In conventional methods, the Least Squares Estimation (LSE) is used. LSE has some advantages such as simplicity of the calculation algorithm. In addition, the properties of the stochastic and functional models do not change from beginning to the end.

#### 2.1.1. Data Snooping

Data Snooping (DS) and other conventional methods use the mean-shift model. In the DS method, it is assumed that only one outlier is present in the observation set. In practice, this method allows detection of more than one outlier and estimation of their locations [3]. DS is performed when the a priori variance of the observation of unit weight is known. The standard deviations of the residuals are calculated using this a priori value. The residuals normalized by this method are normally distributed [4, 5].

#### 2.1.2. Tau Test

If the a priori variance is not known or a value cannot be assigned to it before adjustment, the a posteriori variance $m_{0}^{2}$ produced after adjustment is used for outlier detection.

#### 2.1.3. The t Test

If an observation *l*_i_ includes a gross error *Δ_i_*, using the standard deviation obtained from the invalid adjustment model is not appropriate. In this situation, it is a more accurate approach to compute the $m_{0}^{2}$ value from the residuals that are free from the model errors.

(2) $${\bar{m}}_{0}^{2}=\frac{1}{f-1}(fm_{0}^{2}-\frac{v_{i}^{2}}{{\left(Q_{vv}\right)}_{ii}})$$

where *f* is the degree of freedom, *v* is the vector of residuals, and *Q_vv_* is the cofactor matrix of the residuals.

In Table 1, *P* is the weight matrix of the observations, *s*_0_ is a priori standard deviation of unit weight, *f* is the degree of freedom, *α_0_* is the significance level, *N* represents the normal distribution, *F* represents the Fischer statistic, *χ^2^* represents the Chi-Square statistic, *t* represents the Student (*t*) statistic, and τ represents the Tau statistic. If the correlation among residuals is neglected, the significance level α*_0_* is computed as:

(3) $$\alpha_{0}=1-{\left(1-\alpha \right)}^{1/n}≅\alpha /n$$

where *n* is the number of observations, and a is usually chosen as 5% [7].

### 2.2. Some Robust Estimations

Estimation methods based on LSE are sensitive to the deviations from the normal distribution of observation errors; therefore, LSE is not distributionally robust. We cannot determine that a unique robust method is better than other methods since there is no unique criterion related to the robustness. The most commonly used estimators in the literature are M-, L-, and R-Estimators [8-9]. M-Estimators stand out as the most flexible estimators and considered by many as the most favorable estimator group [8]. M-Estimation is the most convenient technique for debugging observations that include gross errors. It can be applied for heavy tailed normal distribution. It is assumed that the geodetic data follow a normal distribution.

LSE is a special case of M-Estimation, whose score function is $\rho (v)=\overset{n}{\underset{i=1}{\text{∑}}}P_{i}v_{i}^{2}=\text{min}$. The computational algorithm may follow an iteratively reweighted scheme as mentioned in [10-11] although there are other approaches in the literature.

While *ρ*(*v*) is a continuous and convex function, *ψ*(*v*) = *∂ρ*(*v*)/*∂v* is the influence function and *w*(*v*) = *ψ*(*v*)/*v* is the robust weight factor which decreases when the absolute of the residual increases. The estimation procedure is as follows:

(4) $${\bar{P}}_{i}=P\;w_{i-1}$$

(5) $$A^{T}\;\bar{P}\;v=0$$

(6) $$x_{i}={\left(A^{T}\;{\bar{P}}_{i}A\right)}^{-1}A^{T}\;{\bar{P}}_{i}l$$

(7) $$v_{i}=Ax_{i}-l$$

where *P̅* is the equivalent weight matrix, *P* is the first weight matrix, *w* is the robust weight factor, *A* is the design matrix, *v* is the vector of residuals, *x* is the vector of the unknown parameters, *ℓ* is the vector of reduced observations, *i* is the iteration number. In the first iteration, *w* is taken as the unit matrix.

(8) $$w_{0}=I_{n\times n}$$

where *n* is the number of observations. The iterations are executed until the difference between the parameters *x_i_*_+_*_1_* and *x_i_* are negligible. At the end of the sequence of iterations, it is observed that the equivalent weights of the outliers become smaller, even reduced to zero. The weights of the normal observations either do not change during the iterations or show little change.

As shown above, the equivalent weight matrix is used to give a decision about whether observations are normal or outlying. In obtaining the equivalent weight matrix, a robust weight factor is used. The robust weight factors are obtained by comparing the residuals with critical values derived from calculations or given constant values. In order to calculate the critical value, a procedure can be applied as follows:

(9) $$c_{i}=s_{0}\sqrt{Qvv_{ii}}\sqrt{P_{ii}}t_{f,1-\alpha_{0}/2}$$

Here *c* represents the critical value, *s_0_* is the a priori standard deviation, *Qvv* is the cofactor matrix of the residuals, *P* is the weight matrix of the observations, *f* is the degree of freedom, *α*_0_ is the significance level, and *t* represents the Student distribution.

The associate critical value is calculated by averaging the critical values calculated for each observation as:

(10) $$c=\frac{\overset{n}{\underset{i=I}{\text{∑}}}c_{i}}{n}$$

In this study, some of the critical values of the estimations have been calculated and others taken as constant values. The weight functions of the M-Estimations used are listed in Table 2. *s_0_* in Table 2 is the a priori standard deviation of the unit weight, and $v_{i}^{'}=v_{i}/s_{0}$, *ṽ_i_* is the normalized residual which is equal to *v_i_*/*s_vi_*.

### 2.3. Fuzzy Logic Method

In the fuzzy logic approach, the fuzzy set and the statistical test theory are used together. There are two important properties of the fuzzy sets used to identify outliers. These are the complementation and the intersection properties. At the beginning, a statistical test is applied to the residuals and the residuals are classified as ‘normal’ and ‘abnormal’ residuals according to their test statistic [12]. From now on, a test will be called “the first test” when the results are presented:

$$\begin{array}{ll} F\{v_{i}\}:\text{Abnormal residuals subset} & t_{i}>q\\ P\{v_{i}\}:\text{Normal residuals subset} & t_{i}\leq q \end{array}$$

Here *t_i_* shows the test statistic and *q* is the critical value. The membership functions are used to clarify the vagueness concerning the residuals having test statistic very close to the critical value.

(11) $$\mu_{F}(v_{i})=\{\begin{array}{ll} 0 & t_{i}\leq q\\ \frac{1.0}{1.0+{\left(\frac{d}{t_{i}-q}\right)}^{2}} & t_{i}>q \end{array}$$

Here *μ_F_* (*v_i_*) is the membership function related to the subset *F*, d is the standardization component that shows the meaningful deviation magnitude of the test statistic from the critical value [7]. It is impossible to state a definite value for *d*. Therefore, several values have been assigned to this component.

After the definition of the membership values of the residuals, the fuzzy membership relations between the observation errors are determined. The membership values of the residuals and the redundancy matrix are used to realize this goal. The relation between the residuals and the errors is given as follows:

(12) v=−QvvPΔ

Here, the multiplication *Qvv P* is equal to the redundancy matrix *R*, and the equation represents the transformation between the residuals and observation errors. In order to obtain the effects of all observation errors on a residual, the relative redundancy matrix *R̃* is used. The elements of this matrix are obtained as follows:

(13) $$\begin{array}{ll} {\tilde{r}}_{ij}=\frac{\left|r_{ij}\right|}{\underset{j}{\text{max}}(|r_{ij}|)} & \left(i,j=1,2,\ldots ,n\right) \end{array}$$

where *n* is the number of observations, *r_ij_* are the elements of the redundancy matrix, and subscript *j* in the lower position is the column number of the element with maximum value of the *i^th^* row. The rows of the relative redundancy matrix indicate the relative contributions of all observation errors to an individual residual, and the columns indicate the relative contribution of an individual error to all residuals.

(14) $$\tilde{R}=[\begin{array}{cccc} {\tilde{r}}_{11} & {\tilde{r}}_{12} & \cdots & {\tilde{r}}_{1n}\\ {\tilde{r}}_{21} & \underset{\vdots }{{\tilde{r}}_{22}} & \cdots & {\tilde{r}}_{2n}\\ & \vdots & & \\ {\tilde{r}}_{n1} & {\tilde{r}}_{n2} & \cdots & {\tilde{r}}_{nn} \end{array}]$$

The set of the gross errors *H* is defined as the intersection of the set of the errors having the greatest effect on the residuals that are most likely abnormal *A* and the set of the errors having the least effect on the residuals that are most likely normal *B*. The membership value of an observation error in the set *H* is defined as follows:

(15) $$\begin{array}{ll} \mu_{H}(\Delta_{i})=\text{min}(\mu_{A}(\Delta_{i}),\mu_{B}(\Delta_{i})) & \left(i=1,2,\ldots ,n\right) \end{array}$$

In order to obtain the membership values of the observation errors in the set *A* and *B*, the maximum relative contribution of the *i^th^* observation error to the residuals that have membership values equal or greater than 0.5 in the subset *F* and *P* is searched, respectively. Then, this relative value and its complementary value are multiplied by the membership value of the corresponding residual as follows:

(16) $${\tilde{r}}_{ji}=\underset{k=u,v,\ldots ,w}{\text{max}}({\tilde{r}}_{ki})$$

(17) $$\mu_{A}(\Delta_{i})={\tilde{r}}_{ji}\times \mu_{F}(v_{j})$$

(18) $${\tilde{r}}_{mi}=\underset{k=x,y\ldots ,z}{\text{max}}({\tilde{r}}_{ki})$$

(19) $$\mu_{B}(\Delta_{i})=(1.0-{\tilde{r}}_{mi})\times \mu_{P}(v_{m})$$

The membership values of the observation errors in the intersection set *H* are compared with a critical value. This value can be calculated using an arithmetic or weighted mean [7].

Let the number of the elements belonging to set *H* with membership values different from zero be *k*. Now, the critical value *C_μH_* can be calculated with an arithmetic mean as:

(20) $$\begin{array}{ll} C\mu_{H}=\frac{\sum \mu_{H}(\Delta_{i})}{k}=\frac{\left[\mu_{H}(\Delta_{i})\right]}{k} & \mu_{H}(\Delta_{i})\neq 0 \end{array}$$

In the weighted mean method, weights are given to the membership values taking into consideration the relative effect of the observation errors in their own set as follows:

(21) $$P\Rightarrow \{\begin{array}{lll} p_{i}={\tilde{r}}_{ji} & if & \mu_{H}(\Delta_{i})\in \mu_{A}(\Delta_{i})\\ p_{i}=1-{\tilde{r}}_{mi} & if & \mu_{H}(\Delta_{i})\in \mu_{B}(\Delta_{i}) \end{array}$$

(22) $$C\mu_{H}=\frac{\sum p_{i}\mu_{H}(\Delta_{i})}{\sum p_{i}}=\frac{\left[p_{i}\mu_{H}(\Delta_{i})\right]}{\left[p_{i}\right]}$$

After obtaining the observation errors that have membership values greater than the critical value *C_μH_*, the a priori knowledge about the location of these errors is also obtained. In order to verify this determination, a procedure is proposed by [12] as follows:

(23) $$\text{H}=[\begin{array}{cccc} 0 & 0 & \cdots & 0\\ 0 & 1 & \cdots & 0\\ 0 & 0 & 0 & 0\\ \vdots & \vdots & \vdots & \vdots \\ 1 & 0 & \cdots & 0\\ 0 & 0 & \cdots & 1 \end{array}]$$

(24) $$Pss=H^{T}PH-H^{T}PA{\left(A^{T}PA\right)}^{-1}A^{T}PH$$

(25) $$\nabla s=-Pss^{-1}H^{T}Pv$$

where *H_n_*_×_*_m_* is the location matrix of the gross errors, *Pss_m_*_×_*_m_* is the weight matrix of the gross errors, *P_n_*_×_*_n_* is the weight matrix of the observations, *A_n_*_×_*_u_* is the design matrix, *v_nx1_* is the vector of the residuals, ∇*s_mx1_* is the vector of the gross errors. Here, the indices *n* and *m* are the number of all observations and the number of the observations that exceed the critical value *C_μH_*, respectively. In the *H* matrix, the column element that corresponds to the observation with gross error is taken as 1. Consequently, the significance of the estimated gross errors is tested using one of the statistical tests. When presenting the results, this will be the last test.

## 3 Results and Discussion

### 3.1. Experiments and Analyses

In this study, three GPS networks (see Figure 1) have been evaluated to examine outliers using different methods. In order to focus only on the networks and not on the external constraints a free adjustment strategy has been applied. The properties of the networks are listed in Table 3. Various calculation techniques can be used to compute the a priori standard deviation of the unit weight (*s_0_*).

For instance, one of these techniques uses the loop closures. But, this is not correct in some situations. In GPS networks, loop closures are not independent since the same variables are used in neighboring closures, and the weights of the loop closures are not equivalent which are not like those in triangle closures. GPS baseline components are correlated, i.e. every baseline has a *3x3* block of the weight matrix as given in Equation (4). Therefore, a gross error affects all components since weight matrix is non-diagonal. At any rate, the fact that GPS baseline components are correlated makes the detection of possible outliers a question of further research so as to determine whether the corresponding 3D-baseline determination is an outlier as a whole or not. Besides, statistical tests such as the τ *-*test are not rigorously valid (though widely applied) for the case of correlated observations [19]. Since it is crucial to determine*s_0_*, the formula using the median of the absolute values of the residuals with weights is more convenient [18]:

(26) $$s_{0}=\text{med}\{|\sqrt{P_{i}}v_{i}|\}/0.6745$$

where “*med*” denotes median, *P_i_* and *v_i_* are the weight and residual of the observation *ℓ_i_*, respectively. After *s_0_* is obtained, it should not be changed in the iteration steps of the robust estimation.

Conventional methods have been applied to the three networks with two different significance levels of: 0.01, and 0.001. When the significance levels are calculated using Equation (3), a value smaller than 0.001 is obtained for each network. The smaller the significance level, the less sensitive the statistical test to the outliers. In other words, few or no outlier can be determined at small significance levels. Therefore, the smallest significance level is taken as 0.001 for all three networks.

### 3.2. Statistical Tests and Fuzzy Logic Approach for Outlier Detection

In the conventional methods, no outlier has been detected at a significance level of 0.01 for the first and second GPS network (Table 4). Hence, the statistical tests have not been applied at a significance level of 0.001.

As shown in Table 4, the greatest test statistic of the residuals is smaller than the critical value. As a consequence, it is not possible to determine any outlier using the fuzzy logic method since no residual has been named an ‘abnormal’ residual in the first test.

In the conventional methods at a significance level of 0.01, 13 identical observations have been determined as outliers with Tau and the t test for the third GPS network. In DS, there are only two outlying observations as shown in Table 5. In addition, at a significance level of 0.001, DS detected only one observation. The Tau and t tests indicate two outliers that are the same as the results of DS at a significance level of 0.01.

In order to see the changes in the results of the fuzzy logic method, the components of this method have been used alternately. The different applications of the fuzzy logic method are shown in Table 6. Here, the Tau test was applied to the observations at the beginning and at the end. Unlike the first two GPS networks, it was possible to separate the residuals as ‘normal’ and ‘abnormal’ in the third GPS network. So it has been possible to execute the fuzzy logic method. When attention is paid to the results, it can be seen that they are quite compatible with the results of the conventional methods.

### 3.3. Comparison of Some Robust Estimators

We followed only the iteratively reweighted least squares scheme. In robust methods, the critical value has been calculated from equations (9) and (10), and the constant value given is 2 *s_0_* except for Yang-I and Yang-II M-Estimations. As shown in Table 2, the weight functions of Yang-I and Yang-II M-Estimation methods are derived differently. The robust methods with constant critical values are denoted with a superscript star in Table 7.

After applying the robust estimators to the three GPS networks, it has been seen that Danish and Huber methods yield similar results reducing the weights of the suspicious observations during the iterations. Tukey, Andrews, and Yang-I M-Estimations resemble each other reducing the weights to zero. On the other hand, the Yang-II M-Estimation is the method yielding the results that best fit with the statistical test methods and fuzzy logic approach. The methods except for Yang-II M-Estimation produce much more outlying observations when compared with the statistical tests.

Table 7 has been arranged to compare the results of the robust estimations. The performances of different robust estimations in the first GPS network coincide with those in the second and the third GPS networks; therefore, as a representative, Table 7 contains only the results for the first GPS network. In Table 7, the constant and calculated critical values, and the observations whose weights change in iterations are given in Table 7.

## 4. Conclusions

In this study, it has been seen that it is appropriate to apply conventional detection tests at a significance level of 0.001 in GPS networks. Using Equation (3), a value was obtained that was smaller than 0.001 for the three networks. But if the conventional methods are used at very small significance levels, these methods tend to mask the outliers. On the other hand, at greater significance level such as 0.01, more outliers appear to exist in the networks. So, the significance level can be selected as 0.001 in GPS networks that have too many observations. In the first and second GPS networks, there appeared no outliers at any significance level. The opportunity to compare various conventional methods has been taken within the third GPS network. The t and Tau tests indicate the same results at different significance levels. One can be substituted for the other one. DS is different from these two tests and denotes few outliers. This behavior of DS is related to the a priori variance of the networks. If this a priori value is calculated from Equation (26), it is more appropriate to apply DS to the networks instead of other conventional methods.

In the fuzzy logic method, statistical tests have an important effect on the results. When compared with the conventional methods, few outliers are visible with the fuzzy logic method since outliers maintain their effects on the adjustment model throughout the iterations in the conventional methods. Unlike the conventional methods, no observation is removed from the network and the shape of the network is kept to the end in the fuzzy logic method. This characteristic can be seen as an advantage. However, the abundance of the parameters used in this method makes it difficult to use this method as commonly as the conventional methods. In this study, it has been seen that if appropriate values can be given to these parameters, the results are more reliable than the conventional methods. Even if the statistical tests are applied at greater significance level, a more reliable decision can be given about the outliers than the conventional methods.

In robust estimation computed as the iteratively reweighted least squares scheme, it is crucial to determine the critical value that is used by the weight functions. In this study, this critical value is both taken as constant and calculated in applying the robust estimators to the GPS networks. Danish method and Huber M-Estimation usually reduce the weights of the suspicious observations, whereas Tukey, Andrews, and Yang-I M-Estimations tend to make the weights zero. These methods may show similar results with the conventional methods that have great significance levels. But, it has been observed in this study that it is not appropriate to choose great significance levels in the GPS networks with many redundant observations. All the robust methods except for Yang-II M-Estimation produce more outlying observations than determined by the conventional statistical test methods. The Yang-II M-Estimation is compatible with the conventional methods that have small significance levels and the fuzzy logic method.

Since GPS baseline components are correlated, a gross error in one component also affects the other components. Therefore, the detection of possible outliers is a question of further research so as to determine whether the corresponding 3D-baseline determination is an outlier as a whole or not. Besides, statistical tests such as the τ-test are not rigorously valid for the case of correlated observations.

## Figures and Tables

**Table 1.** Test statistic and critical values [6].

**Test**	**Test Statistic**	**Critical Value**
DS	$\frac{\left(Pv_{i}\right)}{s_{0}{\sqrt{\left(PQ_{vv}P\right)}}_{ii}}$	$N_{1-\alpha_{0}/2}=\sqrt{F_{1,\infty ,1-\alpha 0}}=\sqrt{\chi_{1,\infty ,1-\alpha_{0}}^{2}}$
Tau	$\frac{\left(Pv_{i}\right)}{m_{0}{\sqrt{\left(PQ_{vv}P\right)}}_{ii}}$	$\tau_{f,-\alpha_{0}/2}=\sqrt{\frac{f\times t_{f-1,1-\alpha_{0}/}^{2}}{f-1+t_{f-1,1-\alpha_{0}/}^{2}}}$
t	$\frac{\left(Pv_{i}\right)}{{\bar{m}}_{0}{\sqrt{\left(PQ_{vv}P\right)}}_{ii}}$	*t_f_*_−_*_1,1_*_−_*α_0_*_/_*_2_*

**Table 2.** The weight functions of the M-estimations.

**M-Estimation**	**Weight Function**	**Critical Value**
Andrews	$w_{i}=\{\begin{array}{cc} sin(v_{i}/c)/v_{i}/c) & \left|v_{i}\right|\leq c\pi \\ 0 & \left|v_{i}\right|>c\pi \end{array}$	*1.5s_0_ -2s_0_*
[13]		
Huber	$w_{i}=\{\begin{array}{cc} 1 & \left|v_{i}\right|\leq c\\ \frac{c}{\left|v_{i}\right|} & \left|v_{i}\right|>c \end{array}$	*1.5s_0_ - 2s_0_*
[10, 14,15]		
Tukey	$w_{i}=\{\begin{array}{cc} {\left[1-{\left(\frac{v_{i}}{c}\right)}^{2}\right]}^{2} & \left|v_{i}\right|\leq c\\ 0 & \left|v_{i}\right|>c \end{array}$	*1.5s_0_ - 2s_0_*
[15]		
Danish	$w_{i}=\{\begin{array}{cc} exp\left(-\frac{v_{i}^{2}}{c^{2}}\right) & \left|v_{i}\right|>c\\ 1 & \left|v_{i}\right|\leq c \end{array}$	*1.5s_0_ - 2s_0_*
[3, 16]		
Yang-I	$w_{i}=\{\begin{array}{cc} 1 & \left|v_{i}^{'}\right|\leq c_{0}\\ \frac{c_{0}}{\left|v_{i}^{'}\right|}{\left(\frac{c_{1}-\left|v_{i}^{'}\right|}{c_{1}-c_{0}}\right)}^{2} & c_{0}<\left|v_{i}^{'}\right|\leq c_{1}\\ 0 & \left|v_{i}^{'}\right|\geq c_{1} \end{array}$	*c_0_* = *1.0 ∼ 1.5*
[17]		*c_1_* = *2.5 ∼ 3.0*
Yang-II	$w_{i}=\{\begin{array}{ll} 1 & \left|{\tilde{v}}_{i}\right|\leq c_{0}\\ \frac{c_{0}}{\left|{\tilde{v}}_{i}\right|} & c_{0}<\left|{\tilde{v}}_{i}\right|\leq c_{1}\\ 0 & \left|{\tilde{v}}_{i}\right|\geq c_{1} \end{array}$	*c_0_* = *2.0 ∼ 3.0*
[18]		*c_1_* = *4.5 ∼ 8.5*

**Table 3.** Properties of the GPS networks.

**Information about networks**	**1^st^ Network**	**2^nd^ Network**	**3^rd^ Network**
Number of the points	8	11	39
Number of the baselines	15	24	148
Number of the observations (*n*)	15x3 = 45	24 × 3 = 72	148 × 3 = 444
Number of the unknowns (*u*)	8x3 = 24	11 × 3 = 33	39 × 3 = 117
Datum defect (*d*)	3	3	3
Redundant observations (*f* = *n-u*+*d*)	24	42	330
Number of the triangles ( *n_t_* )	9	20	214
A priori standard deviation ( *s_0_* ) mm	0.51	0.35	4.36

**Table 4.** Results of the conventional methods for the first and second GPS network.

	**Statistical Test**	**Significance Level**	**Maximum Test Statistic**	**Critical Value**	**Outlier**
**First GPS Network**	Tau	0.01	2.2395	2.4749	-
	DS	0.01	2.0629	2.5808	-
	t	0.01	2.4675	2.8073	-

**Second GPS Network**	Tau	0.01	2.3791	2.5190	-
	DS	0.01	1.5319	2.5808	-
	t	0.01	2.3977	2.7012	-

**Table 5.** Results of the conventional methods for the third GPS network.

**Statistical Test / Significance Level**	**Iteration**	**Maximum Test Statistic**	**Critical Value**	**Outlier Number**	**Outlier**
Tau	1	4.0925	2.5687	330	ΔZ_33-21_
(0.01)	2	3.9428	2.5687	139	ΔX_21-7_
	3	3.0511	2.5686	360	ΔZ_35-17_
	4	3.0559	2.5685	80	ΔY_17-12_
	5	3.3140	2.5684	41	ΔY_12-11_
	6	3.1411	2.5684	7	ΔX_4-3_
	7	3.0854	2.5683	128	ΔY_20-6_
	8	3.3530	2.5682	290	ΔY_31-20_
	9	3.3194	2.5682	146	ΔY_21-20_
	10	3.0014	2.5681	123	ΔZ_20-8_
	11	2.6804	2.5680	265	ΔX_30-3_
	12	2.6504	2.5679	89	ΔY_17-15_
	13	2.8887	2.5678	83	ΔY_17-13_
	14	2.5481	2.5678	-	-
DS	1	4.5172	2.5808	330	ΔZ_33-21_
(0.01)	2	2.8159	2.5808	139	ΔX_21-7_
	3	2.1159	2.5808	-	-
t	1	4.2327	2.5909	330	ΔZ_33-21_
(0.01)	2	3.9380	2.5910	139	ΔX_21-7_
	3	3.1355	2.5911	360	ΔZ_35-17_
	4	3.0966	2.5913	7	ΔX_4-3_
	5	3.1360	2.5914	80	ΔY_17-12_
	6	3.4402	2.5916	41	ΔY_12-11_
	7	3.1186	2.5917	128	ΔY_20-6_
	8	3.4185	2.5919	290	ΔY_31-20_
	9	3.3846	2.5920	146	ΔY_21-20_
	10	3.0328	2.5922	123	ΔZ_20-8_
	11	2.7204	2.5924	265	ΔX_30-3_
	12	2.6503	2.5925	89	ΔY_17-15_
	13	2.8863	2.5927	83	ΔY_17-13_
	14	2.5693	2.5929	-	-
Tau	1	4.0925	3.2710	330	ΔZ_33-21_
(0.001)	2	3.9428	3.2709	139	ΔX_21-7_
	3	3.0511	3.2707	-	-
DS	1	4.5172	3.3003	330	ΔZ_33-21_
(0.001)	2	2.8159	3.3003	-	-
t	1	4.2327	3.3203	330	ΔZ_33-21_
(0.001)	2	3.9380	3.3206	139	ΔX_21-7_
	3	3.1355	3.3209	-	-

**Table 6.** Results of the fuzzy logic method for the third GPS network.

**FT/ SL**	**SC**	**CM and CV**	**Initial Outliers**	**LT/ SL**	**Outliers**
Tau	0.1	AM 0.6893	ΔY_17-12_, ΔY_20-6_, ΔX_21-7_, ΔZ_21-7_, ΔX_33-21_, ΔZ_33-21_	Tau	ΔY_17-12_, ΔY_20-6_, ΔZ_33-21_
(0.01)	0.1	WM 0.6762	ΔY_17-12_, ΔY_20-6_, ΔX_21-7_, ΔZ_21-7_, ΔX_33-21_, ΔZ_33-21_	(0.01)	ΔY_17-12_, ΔY_20-6_, ΔZ_33-21_
	0.05	AM 0.7073	ΔY_17-12_, ΔY_20-6_, ΔX_21-7_, ΔZ_21-7_, ΔZ_33-21_		ΔY_17-12_, ΔY_20-6_, ΔZ_33-21_
	0.05	WM 0.6913	ΔY_17-12_, ΔY_20-6_, ΔX_21-7_, ΔZ_21-7_, ΔX_33-21_, ΔZ_33-21_		ΔY_17-12_, ΔY_20-6_, ΔZ_33-21_
	0.01	AM 0.6893	ΔY_17-12_, ΔY_20-6_, ΔX_21-7_, ΔZ_21-7_, ΔX_33-21_, ΔZ_33-21_		ΔY_17-12_, ΔY_20-6_, ΔZ_33-21_
	0.01	WM 0.6649	ΔY_17-12_, ΔY_20-6_, ΔX_21-7_, ΔZ_21-7_, ΔX_33-21_, ΔZ_33-21_		ΔY_17-12_, ΔY_20-6_, ΔZ_33-21_
Tau	0.1	AM 0.7346	ΔZ_33-21_	Tau	ΔZ_33-21_
(0.001)	0.1	WM 0.7346	ΔZ_33-21_	(0.001)	ΔZ_33-21_
	0.05	AM 0.7346	ΔZ_33-21_		ΔZ_33-21_
	0.05	WM 0.7346	ΔZ_33-21_		ΔZ_33-21_
	0.01	AM 0.7451	ΔX_21-7_		ΔX_21-7_
	0.01	WM 0.7449	ΔX_21-7_		ΔX_21-7_

The abbreviations used in Table 6 are as follows:
FT: First testSL: Significance levelSC: Standardization component of the membership functionCM: Calculation method of the critical value (*C_μH_*)CV: Critical valueAM: Arithmetic meanWM: Weighted meanLT: Last test

**Table 7.** Results of the Robust Estimators for the First GPS Network.

Observation	Robust Estimations									
	c=1.89 mm (calculated)					c*=1.02 mm (constant)				

	Danish	Huber	Tukey	Andrews	Yang-I	Yang-II	Danish*	Huber*	Tukey*	Andrews*
ΔZ_3-2_					☑				☑	☑
ΔX_3-1_	☑	☑	☑	☑	☑		☑	☑	☑	☑
ΔY_3-1_	☑	☑	☑	☑	☑		☑	☑	☑	☑
ΔX_4-3_									☑	☑
ΔY_4-3_								☑	☑	☑
ΔZ_4-3_	☑	☑	☑	☑	☑		☑		☑	☑
ΔZ_5-4_			☑	☑	☑		☑		☑	☑
ΔY_6-5_			☑		☑				☑	☑
ΔZ_6-5_									☑	
ΔX_6-3_	☑	☑	☑	☑	☑		☑	☑	☑	☑
ΔY_6-3_	☑	☑	☑	☑	☑		☑	☑	☑	☑
ΔZ_6-3_	☑	☑	☑	☑	☑		☑	☑	☑	☑
ΔY_7-1_									☑	
ΔX_7-6_									☑	
ΔY_7-6_	☑	☑	☑	☑	☑		☑	☑	☑	☑
ΔZ_7-6_									☑	☑
ΔY_8-7_									☑	
ΔZ_8-7_			☑		☑		☑	☑	☑	☑
